# Metagenomic Profile of the Bacterial Communities Associated with *Ixodes ricinus* Ticks

**DOI:** 10.1371/journal.pone.0025604

**Published:** 2011-10-13

**Authors:** Giovanna Carpi, Francesca Cagnacci, Nicola E. Wittekindt, Fangqing Zhao, Ji Qi, Lynn P. Tomsho, Daniela I. Drautz, Annapaola Rizzoli, Stephan C. Schuster

**Affiliations:** 1 Department of Biodiversity and Molecular Ecology, Research and Innovation Centre, Fondazione Edmund Mach, San Michele all'Adige, Italy; 2 Department of Biochemistry and Molecular Biology, Center for Comparative Genomics and Bioinformatics, The Pennsylvania State University, University Park, Pennsylvania, United States of America; Duke University Medical Center, United States of America

## Abstract

Assessment of the microbial diversity residing in arthropod vectors of medical importance is crucial for monitoring endemic infections, for surveillance of newly emerging zoonotic pathogens, and for unraveling the associated bacteria within its host. The tick *Ixodes ricinus* is recognized as the primary European vector of disease-causing bacteria in humans. Despite *I. ricinus* being of great public health relevance, its microbial communities remain largely unexplored to date. Here we evaluate the pathogen-load and the microbiome in single adult *I. ricinus* by using 454- and Illumina-based metagenomic approaches. Genomic DNA-derived sequences were taxonomically profiled using a computational approach based on the BWA algorithm, allowing for the identification of known tick-borne pathogens at the strain level and the putative tick core microbiome. Additionally, we assessed and compared the bacterial taxonomic profile in nymphal and adult *I. ricinus* pools collected from two distinct geographic regions in Northern Italy by means of V6-16S rRNA amplicon pyrosequencing and community based ecological analysis. A total of 108 genera belonging to representatives of all bacterial phyla were detected and a rapid qualitative assessment for pathogenic bacteria, such as *Borrelia, Rickettsia* and *Candidatus* Neoehrlichia, and for other bacteria with mutualistic relationship or undetermined function, such as *Wolbachia* and *Rickettsiella*, was possible. Interestingly, the ecological analysis revealed that the bacterial community structure differed between the examined geographic regions and tick life stages. This finding suggests that the environmental context (abiotic and biotic factors) and host-selection behaviors affect their microbiome.

Our data provide the most complete picture to date of the bacterial communities present within *I. ricinus* under natural conditions by using high-throughput sequencing technologies. This study further demonstrates a novel detection strategy for the microbiomes of arthropod vectors in the context of epidemiological and ecological studies.

## Introduction

Biological vectors of human and animal diseases are typically arthropod species that are able to transmit infectious agents to humans and to other warm and cold-blooded animal hosts. Among them, ticks transmit the greatest variety of human and animal pathogens of any blood sucking arthropod [1]. The sheep tick *Ixodes ricinus* is the most common tick species in Europe and the primary vector of a broad range of disease-causing bacteria, including *Borrelia burgdorferi* sensu lato (s.l.), the causative agent of Lyme borreliosis [2]. The *B. burgdorferi* s.l. group comprises four *Borrelia* species recognized as pathogenic for humans, *B. burgdorferi* sensu stricto (s.s.), *B. afzelii*, *B. garinii*, and *B. spielmanii. I. ricinus* is also a competent vector of pathogenic bacteria species within *Rickettsia, Anaplasma* and *Ehrlichia* genera [3]. Besides pathogenic agents, other microbes coexist in *I. ricinus*, such as endosymbionts, commensals or microbes acquired from the blood meal on animal hosts [4], [5]. To date little is known about the bacterial community structures in *I. ricinus* under natural conditions. Investigations on complete microbial communities associated with ticks so far have primarily relied on low throughput molecular techniques, such as culture or cloning-based methods, allowing for the characterization of only a fraction of the *I. ricinus* microbiome [6], [7], [8], [9]. With the advent of metagenomic approaches limitations of these methods have been overcome, enabling the identification of entire microbial communities associated with the host. For ecological purposes, high-throughput shotgun sequencing has been applied to the characterization of microbial communities in complex ecosystems, such as soil and ocean water [10], and more recently to the investigation of infectious agents of medical [11], [12] and veterinary importance [13], [14]. In addition, another approach known as 16S rRNA amplicon pyrosequencing has also been employed for the exploration of microbial diversity in environmental samples [15], as well as in healthy and clinical human specimens [16], [17].

The combination of the above methods together with the increasing number of genomic data resources of tick-borne pathogens [18] allows to exploit the taxonomic diversity of the host-associated content of complex biological systems, such as that of *I. ricinus*. In this study, we evaluated the potential of a shotgun metagenomic approach using short-read next-generation sequencing technologies to detect known tick-borne pathogens and to describe the putative core microbiome of unfed adult *I. ricinus* ticks. In parallel, we surveyed and analyzed the bacterial communities in pooled *I. ricinus* (consisting of 20 pools of 5 nymphs and 20 individual adults per site) at different life stages and from different geographic areas by means of V6-16S rRNA amplicon pyrosequencing. The latter approach allowed us to examine the taxa composition of the bacterial communities associated within tick pools by means of multivariate statistical analyses, commonly used in community ecology. Our findings demonstrate the applicability and the high sensitivity of shotgun metagenomic and amplicon pyrosequencing methods as accurate surveillance tools for the identification of medically important pathogens and other associated bacteria within *I. ricinus*, thereby providing useful information of epidemiological and ecological significance.

## Methods

### Ethics Statement

Permissions for tick collection within the studied sites were obtained, when required, by the regional forestry and wildlife offices and by the administration of the National Park Dolomiti Bellunesi. For national regulation no formal permit for tick collection is required.

### Tick sample collection

Host-seeking ticks were collected in 2006 from vegetation by dragging a 1 m^2^ white blanket in areas of Northern Italy where tick-borne pathogens, such as *B. burgdorferi s.l.* and *A. phagocythophilum* are known to be endemic [19], [20]. Field-collected ticks were microscopically identified as *I. ricinus* and classified by life stage (nymph or adult) using standard taxonomic keys [21]. Live ticks were subsequently washed once in 70% ethanol and deionised water for five minutes each to remove environmental contaminants. *I. ricinus* ticks were grouped according to life stage (nymphs were pooled in groups of 5, while adults were stored individually) and then stored at −20°C in RNAlater® (Ambion, Austin, USA) until nucleic acid extraction. *I. ricinus* ticks subjected to amplicon pyrosequencing were randomly selected among those collected during 2006 (Table S1) in two distinct geographical regions characterized by different tick-borne pathogen infection risk levels for humans (TN Region: Trento Province, Lamar site; BL Region: Belluno Province, Candaten site), as well as by dissimilar ecological features, such as forest and animal host compositions [22].

### Preparation of genomic DNA, total RNA and cDNA from single adult I. ricinus

*I. ricinus* adult ticks were individually subjected to the extraction of genomic DNA and total RNA by means of the All-Prep DNA/RNA Mini Kit according to the manufacturer's protocol (Qiagen, Hilden, Germany). Individual ticks were first ground in 350 µl of Buffer RLT Plus with 5 mm stainless steel beads using a tissue lyser (Qiagen, Hilden, Germany). The genomic DNA and the total RNA were eluted in 80 µl of Buffer EB and 30 µl of RNase-Free water, respectively. Double-stranded cDNA was amplified from the total RNA (0.5 µg) of an unfed female adult *I. ricinus* (Ir 1-4) using the SMART PCR cDNA Synthesis kit (Clontech) (Mountain View, CA) after removal of contaminating genomic DNA using the Turbo DNA-free Kit (Applied Biosystems/Ambion, CA).

### Conventional PCR and phylogenetic analysis

Genomic DNA extracted from adult *I. ricinus* ticks was tested for *B. burgdorferi* s.l. infection by means of a touch-down PCR protocol [23] targeting a 377-bp DNA fragment in the intergenic spacer region (IGS) between genes encoding the 5S and 23S rRNA subunits using primers that have been previously described [24]. Amplified PCR products were subjected to conventional Sanger sequencing. In addition to sequences obtained from this study, a set of representative homologous sequences of *Borrelia* spp. were gathered from a previous study [25] and included in the phylogenetic analysis. The sequence alignment was created using the MUSCLE program [26]. The best nucleotide substitution model for the dataset was estimated using the Akaike information criterion implemented in the Modeltest v3.7 [27] and a Bayesian Markov chain Monte Carlo method implemented in the software MrBayes v3.1.2 [28] was used to construct phylogenetic trees and to assess statistical support for the clades.

### Roche-454 GS FLX pyrosequencing and data analysis

Genomic DNA and cDNA generated from the single adult tick Ir 1-4 were subjected to preparation of single-stranded DNA (ssDNA) libraries and subsequently to pyrosequencing on a Roche GS FLX sequencer (Roche Applied Sciences/454 Life Sciences, Brandford, CT) as previously described [29]. In total, three runs on a quarter PicoTiterPlate™ were performed for the tick sample Ir 1-4: two runs for the genomic DNA library and one run for the cDNA library. The raw sequences generated from the two 454 runs on the Ir 1-4 genomic DNA were combined for further analyses. The unassembled read sequences obtained from these 454 runs were filtered for DNA repeats using RepeatMasker (http://www.repeatmasker.org) and then compared to the NCBI non-redundant protein database (http://www.ncbi.nlm.nih.gov) using BLASTX (protein homology) (*e-*value <1×10^−5^) [30]. The resulting BLAST alignments were analyzed and assigned to taxa in the NCBI taxonomy database using MEGAN v3.9 [31] with the following parameters: MinScore cutoff ≥40, TopPercent: 10, MinSupport: 1.

### Illumina GAIIx sequencing and data analysis

The genomic libraries for the Illumina sequencing were generated from two tick samples naturally infected by *Borrelia* spp., Ir 13-4 (adult male) and Ir 20-8 (adult female), according to the Illumina paired-end library preparation protocol (Illumina, San Diego, CA, USA). The average size of the library fragments was 329 bp while the average of the insert size was 200 bp. The libraries generated were subsequently denatured to obtain ssDNA library fragments and diluted to a final concentration of 7.7 pM each. One hundred and twenty microliters of each ssDNA library was loaded into single lanes of the flow-cell for cluster generation, which was performed on the Illumina Cluster Station according to the manufacturer's instruction (Illumina, San Diego, CA, USA). Paired-end sequencing was performed on the Illumina Genome Analyzer IIx in various sequencing runs.

In order to identify bacterial microorganisms harboured by the single ticks, we mapped the sequence data (76-bp paired-end Illumina reads) from above runs using BWA v0.5.8a [32] with default parameters to a custom database containing all currently available sequenced bacterial genomes (a total of 1242 complete bacterial genomes deposited in GenBank and downloaded from NCBI on August 2, 2010). Lastly, inGAP v2.5.0 [33] was used to graphically visualize the mapped datasets against reference genomes.

### Preparation of genomic DNA and V6-16S rRNA amplicon libraries

Twenty pools consisting each of five nymphs (N = 100) and 20 individual adults were randomly chosen per geographic region for the amplicon pyrosequencing (field-collected ticks from two different geographic regions in Northern Italy (see above)). The tick samples were ground in 285 µl of phosphate-buffered saline (PBS) (Sigma, St. Louis, USA) with 5 mm stainless steel beads using a tissue lyser (Qiagen, Hilden, Germany). Genomic DNA was extracted by using the Blood and Tissue Kit (Qiagen, Hilden, Germany) from 100 µl tick homogenate following the manufacturer's recommended protocol.

Presence of bacterial microorganisms was tested on 200 nymphal and 40 adult *I. ricinus* ticks using barcode-tagged primers that specifically amplify the hypervariable region, V6, of the16S rRNA gene [34]. The composite primers consisted of 454-specific A and B adaptors at their 5′-end, which are required for the 454-conal amplification and pyrosequencing reactions, four-base barcodes tagging the geographic origin of the tick samples, and specific primers for the V6 hypervariable region. The latter primers were designed manually on the conserved stretches of sequence flanking the V6 hypervariable region based on the multiple alignment of the 16S rRNA gene sequences of bacteria of interest (V6-F: 5′ CGCACAAGCGGTGGAGCAT 3′ and V6-R: 5′ TCGTTGCGGGACTTAACCCAAC 3′) (nt- 872-1052 of the complete 16S rRNA gene of *Escherichia Coli*, GenBank accession no. AJ605115). Using the tick genomic DNA as templates, the FastStart High Fidelity PCR System (Roche, Switzerland) was utilized for all the PCR reactions generating the V6 amplicons. The manufacturer's recommended protocol for the Amplicon Preparation (Roche Manual) was used for the PCR reaction mix. The thermal profile for the PCR reactions was: 94°C for 3 min: 32 cycles of 94°C for 30 sec, 56°C for 45 sec, 72°C for 2 min, and a final extension at 72°C for 2 minutes. Each amplicon product was purified using AMPure beads (Agencourt Bioscience Corporation, MA, USA) and subsequently quantified using PicoGreen double-stranded DNA (dsDNA) reagents (Invitrogen Life Technologies, CA, USA). The dsDNA amplicon libraries were combined at equimolar concentrations in two pools, nymph and adult ticks, respectively, and subjected to clonal amplification by means of emulsion PCR followed by pyrosequencing on a 454 GS FLX sequencer (half plate per pool).

### V6 -16S rRNA amplicon 454-pyrosequencing data analysis

The sequence reads obtained from the pyrosequencing of the two pools of amplicon dsDNA libraries generated from nymphal and adult ticks were subsequently associated with their geographic origin using a Python script (available on request) according to their 5′-end barcodes. This resulted in a total of four datasets according to the tick's life stage (nymph and adults) and geographic region (TN Region and BL Region). After trimming the barcodes and primers from the raw sequences, each dataset was compared to the Ribosomal Database Project (RDP) version 10 (http://rdp.cme.msu.edu/) [35]. The filtered reads were analyzed using the Naïve Bayesian rRNA Classifier version 2.0 (RDP-classifier), which provides a taxonomical hierarchy classification (from domain to genus) of bacteria based on the similarity to the complete or partial 16S rRNA gene along with a bootstrap confidence estimates [17]. The output results were further analyzed and compared using MEGAN v3.9 software.

### Ecological analysis of bacterial communities assessed in tick pools

The bacterial communities detected in tick pools were compared between life stages within the same geographic site and among geographic sites using descriptive indexes of niche breadth and overlap. The standardized Levins index B*_sta_*, was applied to compare the number of bacterial taxa (bacterial taxonomical rank  =  genus), and the Pianka index O, to assess the actual overlap in bacterial taxa composition between communities [36]. Both indexes are comprised between 0 (minimum breadth and overlap, respectively) and 1 (maximum breadth and overlap, respectively).

The general structure of bacterial taxa communities was examined by Detrended Correspondence Analysis, DCA [37], in combination with canonical ordination analysis, and particularly canonical correspondence analysis, CCA, since the bacterial taxa composition dataset reported occurrence, but not abundance of taxa [38]. In CCA, the ordination axes for displaying the taxa matrix are constrained to be linear combinations of the columns of the environmental matrix, in this case represented by tick life stages and geographic regions. The goodness of fit of the biplots was used to evaluate the fraction of variance of all covariances between bacterial taxa and environment accounted for by the diagram [39]. Canonical coefficients were used to assess the influence of environmental variables in structuring the ordinations [40]. All analyses were performed by the software R 2.9.1 (Vegan package).

## Results

### Pathogenic and host-associated bacteria in single I. ricinus ticks

**Figure 1:**
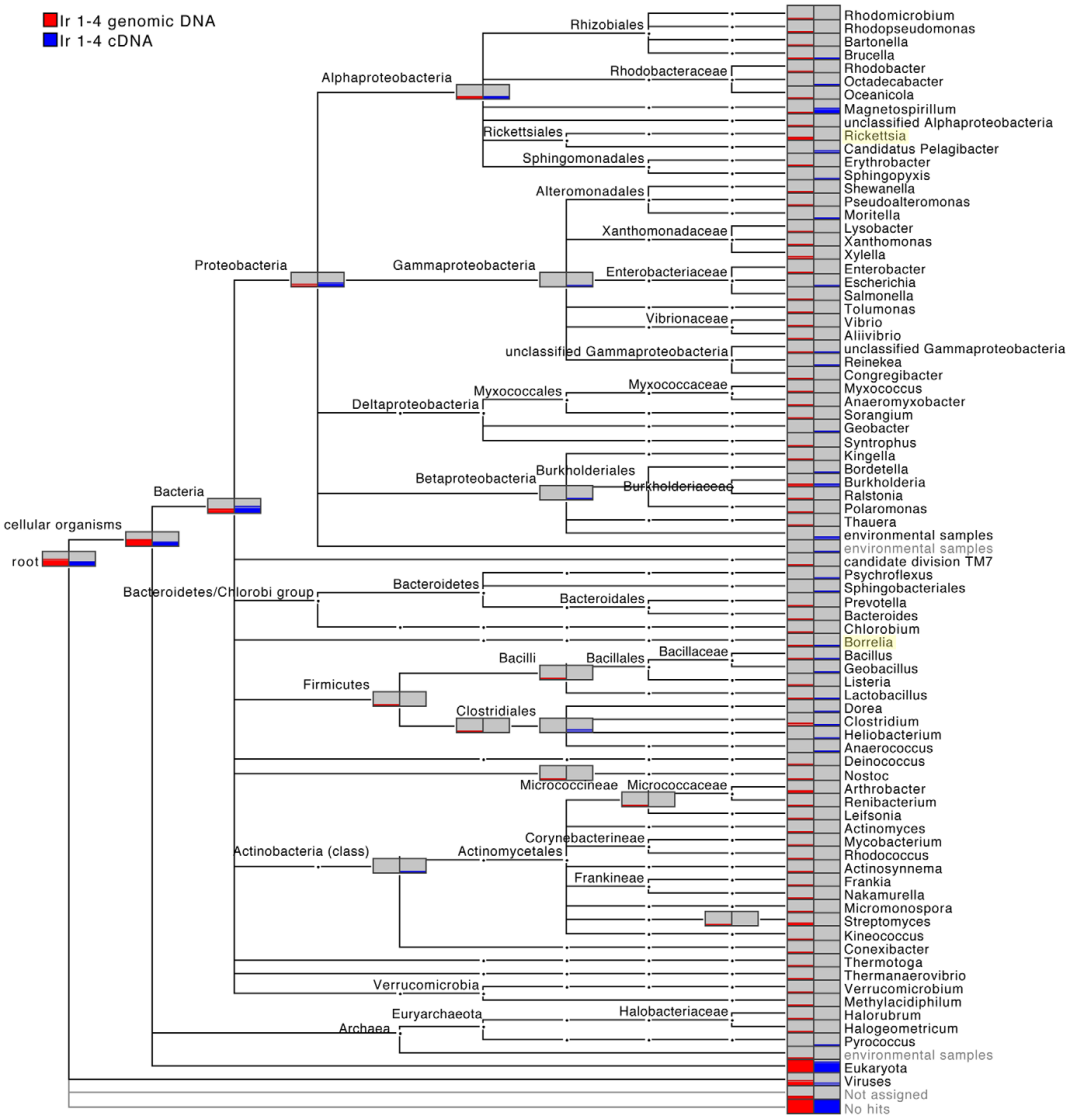
MEGAN comparison of the bacterial taxonomic profiles of genomic DNA (red) and cDNA-derived sequences (blue) from tick sample Ir 1-4. The height of the bars corresponds to the number of hits for each genus. ‘Not assigned’ indicates sequences matching to sequences in the NCBI database that are not assigned to taxa. ‘No hits’ indicates reads for which no sequence match was found in the BLASTX analysis.

Three unfed adult *I. ricinus* ticks (Ir 1-4, Ir 13-4, Ir 20-8) were shown to be naturally infected by different pathogenic *Borrelia* spp. by means of conventional PCR and phylogenetic analysis (Table S1, Figure S1) and were subsequently subjected to a shotgun metagenomic approach. Roche 454 pyrosequencing of ssDNA libraries, obtained from both genomic DNA and cDNA of the single unfed adult tick Ir 1-4, resulted in 127,974 and 60,186 reads, respectively (Table 1). Analysis of the cDNA obtained from the total RNA of the tick was performed to identify protein-coding region and ribosomal RNA transcripts, which support the occurrence of viable and replicating microorganisms. A BLASTX based MEGAN analysis of the genomic DNA- and cDNA-derived sequence datasets revealed assignment of 8.6% and 5%, respectively, of sequence reads to known taxa (bit score cutoff ≥40) based on the homology at protein level (Table S2). 96.6% and 93%, respectively, of the assigned sequence reads matched to Eukaryota, primarily the *Ixodes* genus and other closely related arthropods. Of the assigned reads, 1.1% of the genomic DNA and 0.3% of the cDNA-derived sequences were of bacterial origin, based on coding sequences. A comparative taxonomic profile of the bacterial content at the genus level for the genomic DNA and cDNA is shown in Figure 1.

**Table 1:** 454 GS FLX and Illumina GAIIx sequencing statistics of three single unfed adult *I. ricinus* ticks and number of sequence reads matching to reference genomes.

Tick sample	Type of nucleic acids	Sequencing statistics	Reference genomes	No. of reads matching to ref. genomes
Ir 1-4	Genomic DNA	454 GS FLX (2 quad)	*B. burgdorferi s.s.*	1^a^
		Total no. reads: 127,974	*B. azelii*	0
		Average read length: 159.8 bp	*B. garinii*	0
Ir 1-4	cDNA	454 GS FLX (1 quad)	*B. burgdorferi s.s.*	0
		Total no. reads: 60,186	*B. azelii*	1^a^
		Average read length: 227.4 bp	*B. garinii*	0
Ir 13-4	Genomic DNA	Illumina GAIIx (1 lane)	*B. burgdorferi B31* b	393^c^
		Total no. reads: 56,657,258	*B. azelii PKo* b	652^c^
		Read length: 76 bp^e^	*B. garinii PBi* b	4,652^d^
			*B. garinii PBr* b	4,726^d^
Ir 20-8	Genomic DNA	Illumina GAIIx (1 lane)	*B. burgdorferi B31* b	112^c^
		Total no. reads: 46,555,380	*B. azelii PKo* b	2,313^d^
		Read length: 76 bp^e^	*B. garinii PBi* b	114^c^
			*B. garinii PBr* b	NA

a Reads identified by the comparison against the NCBI non-redundant protein database using BLASTX and assigned to taxa by MEGAN. Bit score cutoff ≥40.

b GenBank accession number of the four chromosome genomes used as reference genomes: *B. burgdorferi B31* NC_001318, *B. afzelii PKo* NC_008277, *B. garinii PBi* NC_006156 and *B. garinii PBr* WGS ABJV00000000.

c Number of reads mapped to the reference genomes by BWA (mapping quality score ≥15).

d Number of reads mapped to the reference genomes by inGAP.

e Paired-end reads.

The identification of bacterial taxa in the tick sample Ir 1-4 was based primarily on unique reads. Heterogeneous findings emerged from the comparison of genomic DNA and cDNA. *Proteobacteria* was found to be the overall dominant bacteria phylum in both data sets, followed by *Actinobacteria* (found predominately in genomic DNA) and *Firmicutes*. Among the bacterial tick-borne pathogens, we found sequences that matched coding regions of the *Borrelia* genus, in both genomic DNA- and cDNA-derived sequences. This finding confirms not only the previous PCR results, but also shows that this pathogen was transcriptionally active in the analyzed tick sample. In addition, in the genomic library we found coding regions belonging to the *Rickettsia* genus. Members belonging to the *Rickettsia* genus are mostly known as arthropod-vectored pathogens, but some are recognized as arthropod endosymbionts, as they have been found exclusively in arthropods without any other known secondary hosts [41]. Other microorganisms detected in the tick Ir 1-4 comprised environmental soil and water bacteria, such as *Magnetospirillum*, *Sorangium, Bacillus, Frankia*, *Clostridium* and *Streptomyces.* Additionally, we identified animal pathogenic bacteria, such as *Bartonella*, *Brucella, Bordetella*, *Burkholderia*, *Mycobacterium* and *Rhodococcus*.

Although a rigorous surface washing of each tick was performed prior any further analysis, some of bacterial genera herein detected are commonly found in soil, on the surface of plants or occasionally associated to the insect gut. We cannot exclude that bacteria detected in low abundance might be part of the tick exoskeleton.

**Figure 2:**
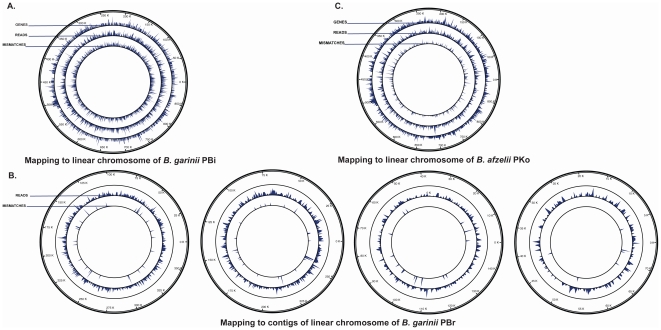
inGAP mapping of genomic DNA-derived sequences obtained from single *I. ricinus* ticks (Illumina paired-end reads) to the reference chromosomes of pathogenic *Borrelia* species. For the purpose of presentation the linear chromosomes of *Borrelia* spp. are circularized. The outer circle designed as ‘READS’ shows the coverage plot over the reference chromosome, while the inner circle displays the amount of estimated nucleotide mismatches. (A). Genomic DNA-derived sequences from Ir 13-4 mapped to the *B. garinii* PBi chromosome (NC_006156). (B) Genomic DNA-derived sequences from Ir 13-4 mapped to the four contigs of the *B. garinii* PBr chromosome (WGS ABJV00000000). (C) Genomic DNA-derived sequences from Ir 20-8 mapped to the *B. afzelii* PKo chromosome (NC_008277).

A different shotgun metagenomic approach was applied to the two tick samples Ir 13-4 and Ir 20-8 using the next-generation sequencing platform Illumina GAIIx. Genomic DNA sequencing of Ir 13-4 and Ir 20-8 resulted in approximately 57 million and 47 million paired-end 76-bp reads, respectively (Table 1). Genomic DNA-derived sequences of the two individual ticks were taxonomically profiled using a computational approach based on the BWA algorithm [32]. Total read numbers of 11,696 and 3,675, respectively, for Ir 13-4 and Ir 20-8, were of bacterial origin. The bacterial diversity and the amount of sequences assigned to each bacterial genus varied between the two single ticks analyzed. With regard to disease-causing bacteria, *B*. *burgdorferi* s.l. was detected at the strain level in both ticks. In particular, *B. garinii* PBr strain was identified in Ir 13-4, (Table 1, Figure 2A, 2B) and *B. afzelii* PKo strain in Ir 20-8 (Table 1, Figure 2C). The Illumina paired-end reads were found to map evenly distributed to the entire *B. garinii* PBr and *B. afzelii* PKo reference genomes, strongly supporting the presence of these pathogenic strains in the tick samples. Moreover, when comparing the Ir 13-4 sequence data with the chromosomal sequence of *B. garinii* PBr, the amount of nucleotide mismatches found corresponded approximately to the predicted error estimated from the Phred quality score of the bases. In comparison, mapping the Ir 13-4 reads with the chromosomal sequence of the *B. garinii* PBi strain, revealed approximately 47X more nucleotide mismatches (Figure 2A, 2B).

Among bacteria genera that have been previously associated with Ixodid ticks, we found genomic DNA-derived sequences in Ir 13-4 and Ir 20-8 samples belonging to *Pseudomonas, Erwinia*, and *Bacillus* [42], [43], [44]. Members of the latter genera inhabit a broad range of environments, including soil, water and insect guts, and some of them can act as opportunistic pathogens of infection in both animals and humans. Interestingly, we detected DNA-derived sequences from all single ticks belonging to *Mycobacterium*, a bacterial genus which includes several human and animal pathogenic species. Non-pathogenic microorganisms identified in this study included commensal bacteria of the gastrointestinal tract and skin, such as *Propionibacterium* and *Bacteroides*. Among bacteria genera detected only in either one of the examined tick samples were *Methylobacterium, Stenotrophomonas, Rhodococcus* and *Bifidobacterium*.

### Assessment of the bacterial communities in pooled ticks using V6-16S rRNA amplicon pyrosequencing

The pyrosequencing approach targeting the V6 hypervariable region of the 16S rRNA gene enabled us to explore and characterize the bacterial diversity in *I. ricinus* tick pools. We analyzed ticks from two different geographic regions in correspondently assembled pools of nymphal and adult ticks. This analysis was performed on pooled ticks in order to maximize the likelihood to detect pathogenic bacteria, whose infection prevalence in tick populations varies in time and in space due to a complex of ecological factors [45].

The V6 target region was chosen because it allows discrimination between most pathogenic and non-pathogenic bacterial taxa [34]. 454 pyrosequencing of V6 amplicon libraries generated 281,327 and 265,778 sequence reads for the nymphal and adult tick pools, respectively (Table S3). Sequence barcoding allowed for discrimination of amplicon reads derived from each geographic region (Table S4).

**Figure 3:**
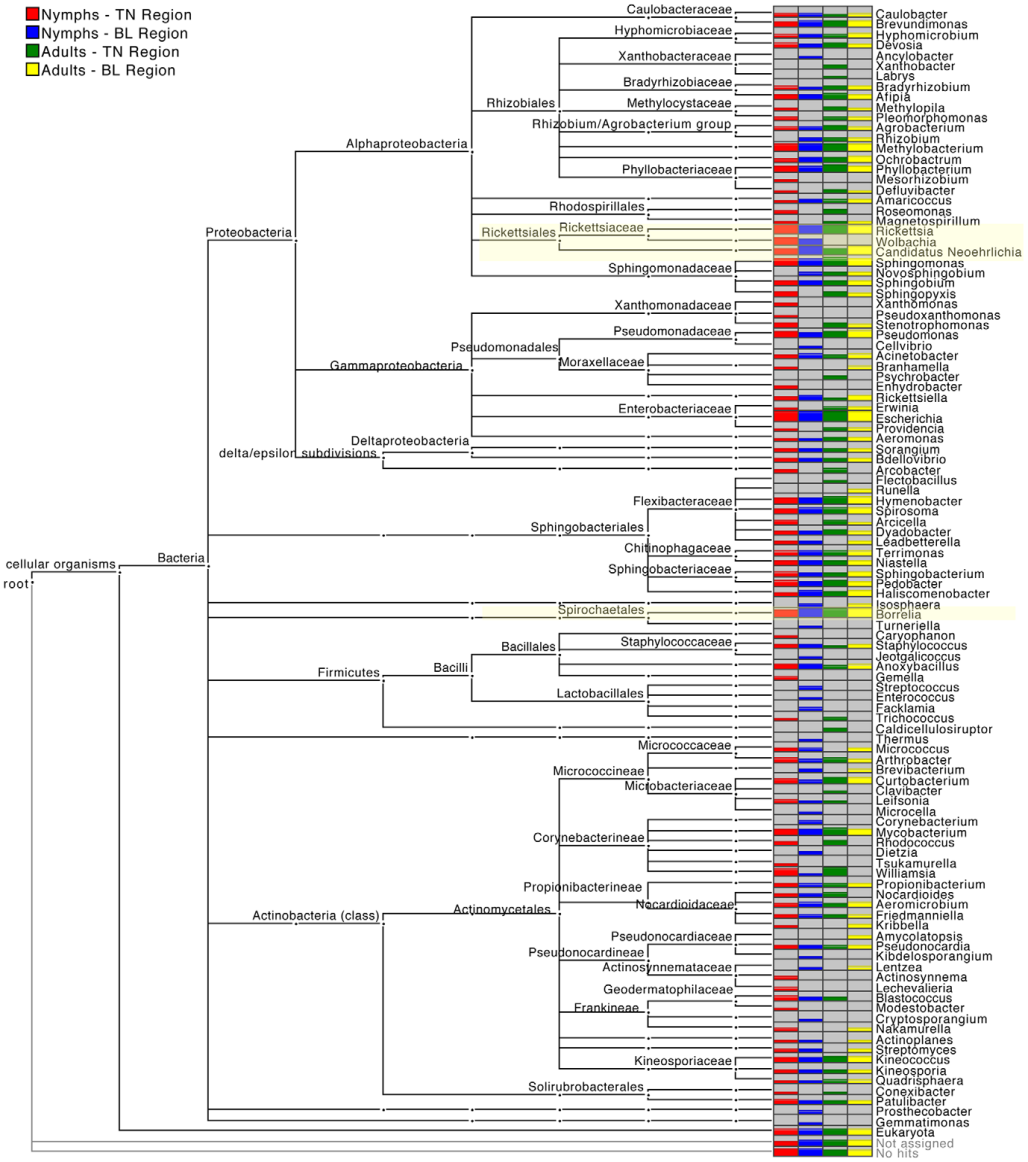
MEGAN comparison of bacterial taxonomic profiles of four tick pools (reflecting different life stages and geographic provenience) based on the V6 amplicon 16S rRNA gene sequence reads analyzed against the Ribosomal Database. The height of the bars corresponds to the number of hits for each genus. Highlighted in light yellow are bacteria genera recognized as tick-borne pathogens or tick endosymbionts.

The taxonomical composition of the four obtained datasets showed *Proteobacteria* as dominant bacterial phylum, followed by the *Actinobacteria, Spirochaetes, Bacteroidetes* and *Firmicutes* phyla, with a total of 108 identified genera, as depicted in Figure 3. The relative abundance of bacterial phyla assessed in the pool ticks is shown in Figure S2.

Among the genera belonging to the *Alphaproteobacteria* class and recognized as medically important arthropod-vectored pathogens, *Borrelia* and *Rickettsia* were the most highly represented in all four pools. *Rickettsia* encompasses both pathogenic and endosymbiontic species, however, the length of the amplicon sequences did not allow for identification of *Rickettsia* on lower taxonomic ranks while keeping a high bootstrap confidence estimate. Interestingly, *Wolbachia* was identified in ticks at the nymphal stage in both geographic regions. Members of the genus *Wolbachia* infect a wide range of arthropod species and are vertically transmitted, causing a variety of reproductive alterations in their arthropod hosts [46]. Importantly, the recently proposed *Candidatus* Neoehrlichia bacteria genus was identified in all four pooled tick samples. This genus includes the *Candidatus* Neoehrlichia mikurensis species, which is a member of the *Anaplasmataceae* family and has been previously reported to infect ticks and wild rodents [47].

Interestingly, V6 ribosomal amplicon sequences were assigned to the genus *Rickettsiella*, which belong to the *Gammaproteobacteria* class, and were identified in both nymphal and adult tick stages, as well as in specimen from both geographic regions. Members of *Rickettsiella* have been recently classified as intracellular bacterial pathogens of arthropods that are capable of affecting stage development and survival of their natural arthropod hosts [48].

Bacterial genera identified by amplicon sequencing also encompassed environmental soil microorganisms like *Methylobacterium*, *Magnetospirillum*, *Sorangium* and *Bdellovibrio* that had not been associated with Ixodid ticks before. Several other bacterial taxa that were detected in the single ticks both by the amplicon pyrosequencing and the shotgun metagenomic approach included *Rhizobium, Pseudomonas, Stenotrophomonas, Erwinia, Mycobacterium* and *Rhodococcus*.

**Figure 4:**
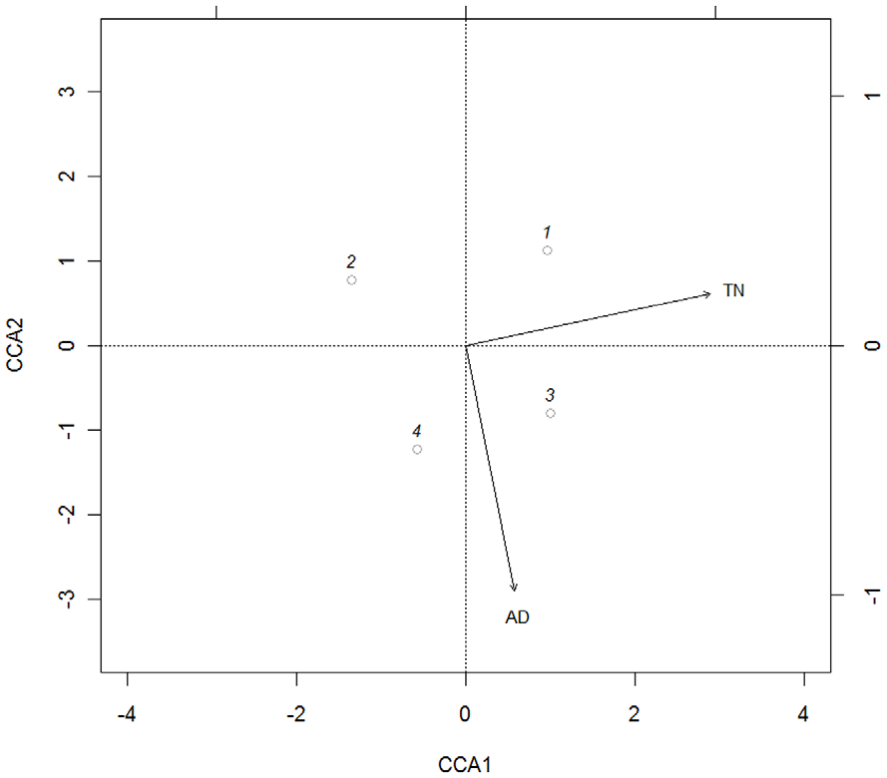
Canonical correspondence analysis ordination diagram. Ordination of sites along the first two canonical axes and the environmental variables, represented by arrows are showed. AD: tick stage, TN: geographic region. Dots represent sites, i.e. 1: nymph ticks, TN region; 2: nymph ticks, BL region; 3: adult ticks, TN region; 4: adult ticks, BL region. Bacterial taxa were omitted for clarity.

While we found a broad bacterial diversity in the examined tick populations based on V6-16S rRNA amplicon sequences, no differences in the occurrence of tick-borne pathogens were observed between tick life stages and geographic regions. The overall bacterial community structure was further evaluated by an ecological analysis. According to the descriptive index of taxa occurrence (index of Levins), the number of bacterial taxa occurring in the two geographic regions was similar, and higher in nymphs than in adults (Table 2). However, the actual overlap (Pianka index) in bacterial taxa occurrence between ticks of the same developmental stage from the two regions was lower than the overlap between nymphal and adult ticks from the same region (Table 2). Therefore, the bacterial taxa composition was more similar between different development stages, than between different geographic regions. Consistent results were obtained through the multivariate community analysis. The DCA first axis eigenvalue was λ = 0.18, and separated the bacterial communities of ticks sampled in the same region (sites scores: 1(nymphs, TN region): −0.55; 2(nymphs, BL region): 0.71; 3(adults, TN region): −0.36; 4(adults, BL region): 0.19), while the second axis was much lower with λ = 5.6*10^−3^, and separated the bacterial communities of the two tick stages (sites scores: 1: −0.55; 2: 0.71; 3: −0.36; 4: 0.19). Consistently, the first axis of CCA (λ = 0.17; Table 3) was mainly a Region axis (biplot score = 0.97; see Table 3 and Figure 4), while the second axis of CCA (λ = 0.13; Table 3) was mainly a Stage axis (biplot score = −0.98; Table 3 and Figure 4). Hence, bacterial communities of ticks sampled in the same region were found to be more similar than those from ticks of the same developmental stage sampled from different regions (Figure 4).

**Table 2:** Taxa occurrence breadth in bacterial community of ticks (Levins index, Bsta) and taxa occurrence overlap (Pianka index, O) between tick stages, and geographic regions. Indexes vary between 0 and 1.

Bsta [0,1]	TN region^a^	BL region^b^	O [0,1]
N tick stage^c^	0.74	0.72	0.76
AD tick stage^d^	0.67	0.69	0.80
O [0,1]	0.83	0.81	

a TN region: Trento Province.

b BL region: Belluno Province.

c N tick stage: Nymphs.

d AD tick stage: Adults.

**Table 3:** Summary of Canonical Correspondence Analysis: Eigenvalues of first two ordination axes, site and biplot scores.

CCA coefficients		CCA1	CCA2
Eigenvalues		0.17	0.13
Site Constrains	Site 1	0.78	1.14
	Site 2	-1.17	0.76
	Site 3	1.20	-0.82
	Site 4	-0.76	-1.20
Constraining variables	AD^a^ (stage)	0.19	-0.98
	TN^b^ (region)	0.98	0.21

a AD: adult tick life stage.

b TN: TN region.

Goodness of fit of biplot is also shown.

## Discussion

This study aims to assess the diversity of bacterial microorganisms associated with *I. ricinus* ticks using high-throughput sequencing techniques. Here we explored for the first time the potential of metagenomics for gathering a comprehensive picture of disease-causing bacteria and host-associated bacteria within their vector, *I. ricinus*.

454- and Illumina-based metagenomic analysis pipelines were successfully applied to detect and characterize the bacterial community residing in whole single *I. ricinus* ticks, without previous separation of the bacterial from the highly abundant host genetic material. The metagenomic approach performed by means of Illumina sequencing technology at a moderate sequencing depth enabled us to identify the causative agent of Lyme borreliosis with high confidence up to the strain level, even without complete genome coverage. Importantly, the *B. garinii* PBr strain was specifically identified, distinctly from *B. garinii* PBi, in the tick sample Ir 13-4. *B. garinii* PBi (also known as OspA serotype 4 strain) has been only recently described as genetically distinct from the *B. garinii* PBr strain and was proposed as a new species, *B. bavariensis* sp. nov. based on the multilocus sequence typing analysis of six chromosomal housekeeping genes [25]. This finding is epidemiologically relevant because *B. garinii* PBi is maintained in nature by an enzootic rodent-tick cycle and is known to be associated with a higher pathogenicity in humans than *B. garinii* PBr. The results indicate that our approach is capable of detecting pathogenic agents with high specificity directly in their biological vectors.

With respect to non-pathogenic bacteria, *Stenotrophomonas, Pseudomonas, Rhodococcus* and *Proprionibacterium* were ubiquitously detected in single *I. ricinus* ticks, as well as in pooled ticks by using amplicon pyrosequencing, suggesting that these microbes might be part of the tick core microbiome. Evidence of the presence of these bacteria in Ixodid ticks have been previously shown by means of traditional molecular methods [8], [42], [43], [49] and more recently in other efforts by means of amplicon pyrosequencing [44].

Our study indicates that shotgun metagenomics provides the foundation for a novel detection strategy for epidemiological studies. More extensive metagenomic studies on single *I. ricinus* ticks will become increasingly accessible thanks to new developments in high-throughput sequencing platforms allowing deeper sequencing at reduced costs and hence the application to a larger sample size. An additional benefit will be the concomitant increase of bacterial genomic databases available for sequence comparisons.

So far, the majority of investigations identifying bacterial diversity in *I. ricinus* ticks was mainly targeted towards pathogenic bacteria and accomplished using low throughput molecular techniques [7]. These techniques have been a limiting factor in evaluating the epidemiological potentials of vectors and their hosted pathogens in their ecological context. Although in this first attempt we only analyzed a few tick individuals, we observed variations in the occurrence of bacterial genera between individual samples. This observation is consistent with previous studies conducted by means of traditional methods and might reflect the ticks' habitats and vector-host interactions [9], [49]. The continuation of the sequencing effort on larger cohorts of single ticks sampled under natural conditions will contribute to identifying patterns of host-microbe association and potential factors affecting the variation of the tick microbiome. For example, future investigations could be applied to assess patterns of microbial co-existence under natural conditions, which could be further tested for microbial interactions under controlled experimental conditions.

By further exploring the bacterial communities associated with *I. ricinus* using V6-16S rRNA amplicon sequencing, we found an even wider diversity of bacterial taxa than with the shotgun metagenomic approach. However, the apparently increased sensitivity to detect members of specific bacterial phyla may be due to a possible amplification bias towards taxa exhibiting higher primer specificity or those that are more abundant in the sample [14], [17]. Notably, this method allowed for a rapid qualitative assessment of pathogenic bacteria, such as *Borrelia, Rickettsia,* and the potential pathogenic genus, *Candidatus* Neoehrlichia. This genus encompasses the *Candidatus* Neoehrlichia mikurensis species, which has been recently reported to cause febrile bacteremia in humans [50], [51], and may represent a new emerging tick-borne pathogen in Europe.

The dominant presence and the significance of *Rickettsiella*-assigned sequence reads in *I. ricinus* ticks requires further investigation considering the close phylogenetic relationship of this genus to vertebrate pathogenic bacteria of the genera *Coxiella* and *Legionella*. Further research is needed in order to clarify whether the bacteria *Rickettsiella* found in our study acts as an arthropod-pathogen or exhibits a mutualistic relationship with its arthropod host. Overall, in combination with barcoding and multiplex 454-pyrosequencing, V6-16S rRNA amplicon sequencing provides a means of surveillance of the circulation of disease-causing bacteria in tick populations from geographical areas not previously explored and of monitoring temporal variations in their circulation. In addition, this method allows for a classic community based ecological analysis to readily be applied to the microbial diversity of an arthropod vector. This approach has the potential to immensely spur microbial ecology applied to arthropod vectors, and its epidemiological counterpart (i.e. investigating pathogen emergence and prevalence in the vectors).

In this study, the community based ecological analysis applied to tick pools showed that the bacterial communities were constrained to their environments. Our results describe a separation according to both geographic area and tick life stage. This finding appears to reflect the different habitats where the ticks were collected in the two geographic regions (and consequently environmental bacteria), and other biotic factors, such as a dissimilar availability of vertebrate hosts, and therefore different animal host-associated bacteria, on which the tick feed. On the other hand, the higher diversity of bacterial taxa observed in the nymphal compared to adult stage may be the result of the distinctive host-selection behavior of immature *versus* mature stages of *I. ricinus*. Indeed, adult *I. ricinus* ticks exhibit a narrow host-selection behavior and feeding preference compared to nymphs (adult ticks feed primarily on vertebrates of larger size, i.e. ungulates, while nymphs feed on smaller mammals and birds as well as larger hosts).

The biological significance of the bacterial diversity and community interactions in arthropods, and especially in *I. ricinus*, is still poorly understood. Previous experimental studies have shown ecological interactions between microbes in ticks and the impact of certain bacterial species on pathogen density and transmission of other tick-borne microbes [49], [52], [53]. Our results show that both shotgun metagenomics and amplicon sequencing approaches can fingerprint the bacterial richness of a microbial community residing in a arthropod vector of human diseases at high resolutions, providing a means of surveillance of pathogen's occurrence in disease vectors from a certain environmental context that contribute to adjust and focus sampling effort on specific hypotheses or epidemiological assessments. Although further research is required to investigate the functional and the ecological implications of the bacterial communities associated with *I. ricinus*, this work represents an initial effort in data acquisition and an innovative analysis towards the characterization of the *I. ricinus* microbiome including disease-causing bacteria under natural conditions.

## Supporting Information

Figure S1 **Unrooted bayesian phylogenetic tree of 92** ***B. burgdorferi*** **s.l. 5S-23S IGS sequences (176-bp).** Tick samples infected by pathogenic *Borrelia* species in this study were: Ir 1-4, Ir 13-4 and Ir 20-8 (Highlighted in colored rectangles). Posterior probabilities of clades are indicated at the nodes. Branches are color-coded based on previously assigned species (according to Margos et al. 2009) as follows: *B. bissettii* - purple, *B. burgdorferi* s.s. - red, *B. garinii/B. bavariensis* - green *B. afzelii* - blue, *B. lusitaniae* - pink *B. valaisiana* - yellow.
(TIF)

Figure S2 **Relative abundance of the major bacterial phyla detected by V6-16S rRNA amplicon pyrosequencing in four** ***I. ricinus*** **tick pools.**
(TIF)

Table S1 **Number of collected host-seeking ticks and prevalence of** ***Borrelia*** **spp. in each sampling site in 2006.**
(DOCX)

Table S2 **Number of genomic DNA- and cDNA-derived sequences of the tick sample Ir-1-4 assigned to major taxonomical nodes by MEGAN analysis after comparison to the NCBI non-redundant protein databases using BLASTX.**
(DOCX)

Table S3 **Properties of 454 Roche GS-FLX pyrosequencing run of the V6-16S rRNA amplicon libraries obtained from two tick pools.**
(DOCX)

Table S4 **Summary of the four datasets of V6-16S rRNA amplicon sequences obtained from the two tick pools after associating their geographic origin with the 5′-end barcodes.**
(DOCX)
