# Supplementary figures and images for: Metagenomic Profile of the Bacterial Communities Associated with Ixodes ricinus Ticks
Source: PLoS One. 2011 Oct 13;6(10):e25604. doi: 10.1371/journal.pone.0025604 (PMC3192763; doi:10.1371/journal.pone.0025604)

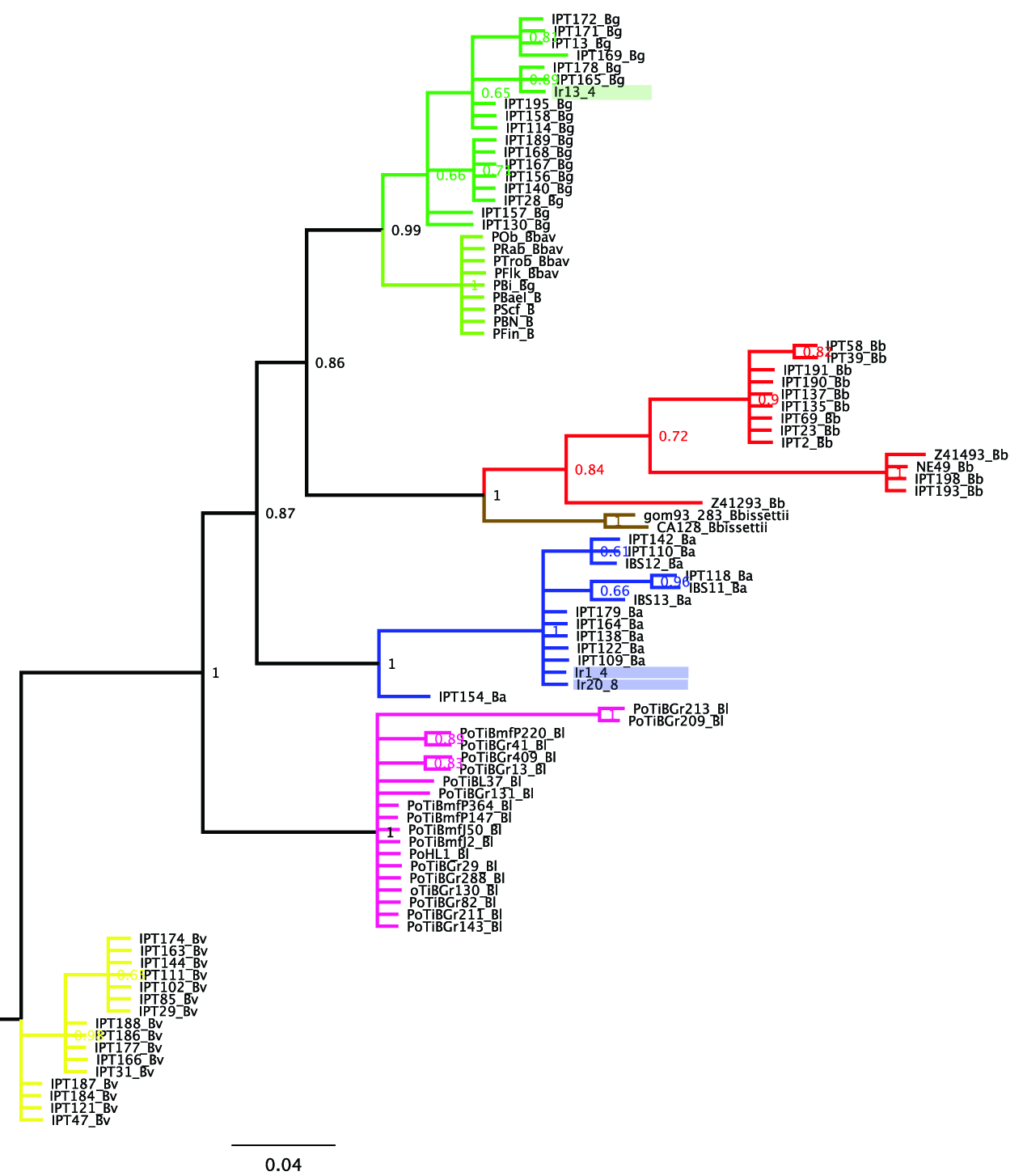

Supplement: Figure S1 — Unrooted bayesian phylogenetic tree of 92 B. burgdorferi s.l. 5S-23S IGS sequences (176-bp). Tick samples infected by pathogenic Borrelia species in this study were: Ir 1-4, Ir 13-4 and Ir 20-8 (Highlighted in colored rectangles). Posterior probabilities of clades are indicated at the nodes. Branches are color-coded based on previously assigned species (according to Margos et al. 2009) as follows: B. bissettii - purple, B. burgdorferi s.s. - red, B. garinii/B. bavariensis - green B. afzelii - blue, B. lusitaniae - pink B. valaisiana - yellow. (TIF) [file pone.0025604.s001.tif]

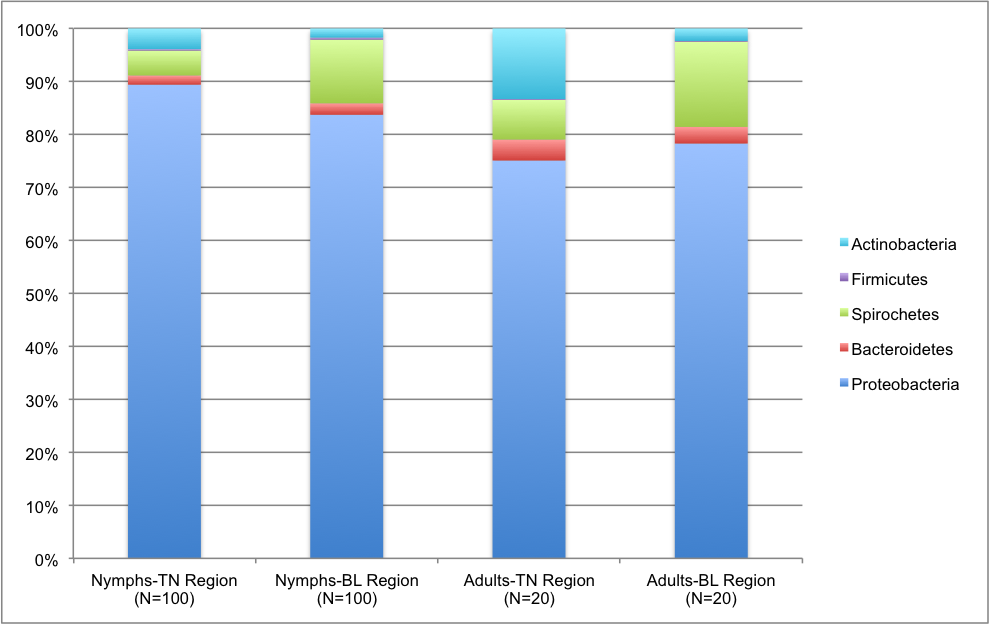

Supplement: Figure S2 — Relative abundance of the major bacterial phyla detected by V6-16S rRNA amplicon pyrosequencing in four I. ricinus tick pools. (TIF) [file pone.0025604.s002.tif]
