# Supplementary material for: Metagenomic Profile of the Bacterial Communities Associated with Ixodes ricinus Ticks
Source: PLoS One. 2011 Oct 13;6(10):e25604. doi: 10.1371/journal.pone.0025604 (PMC3192763; doi:10.1371/journal.pone.0025604)
Supplement: Table S1 — Number of collected host-seeking ticks and prevalence of Borrelia spp. in each sampling site in 2006. (DOCX) [file pone.0025604.s003.docx]

**Table S1**. Number of collected host-seeking ticks and prevalence of *Borrelia* spp. in each sampling site in 2006.

| **Sampling Site** | **Province** | **Total No. host-seeking Nymphs**  **(2006)** | **Total No. host-seeking Adults**  **(2006)** | **No. Pools of**  **Nymphs^a^ tested/positive for**  ***Borrelia* spp.**  **(% Prevalence;**  **95%IC)** | **No. Adults**  **tested/positive for**  ***Borrelia* spp.**  **(%Prevalence;**  **95%IC)** |  |
| --- | --- | --- | --- | --- | --- | --- |
| Lamar | Trento, Italy | 228 | 44 | 41/4 (2.0; 0.7-4.6) | 42/6 (14.3; 5.4-28) |  |
| Candaten | Belluno, Italy | 987 | 156 | 56/13 (5.2; 3-8.1) | 66/12 (18.0; 9.7-29.6) |  |

^a^  Pools of five nymphs each.
