# Supplementary material for: Metagenomic Profile of the Bacterial Communities Associated with Ixodes ricinus Ticks
Source: PLoS One. 2011 Oct 13;6(10):e25604. doi: 10.1371/journal.pone.0025604 (PMC3192763; doi:10.1371/journal.pone.0025604)
Supplement: Table S2 — Number of genomic DNA- and cDNA-derived sequences of the tick sample Ir-1-4 assigned to major taxonomical nodes by MEGAN analysis after comparison to the NCBI non-redundant protein databases using BLASTX. (DOCX) [file pone.0025604.s004.docx]

**Table S2**. Number of genomic DNA- and cDNA-derived sequences of the tick sample Ir-1-4 assigned to major taxonomical nodes by MEGAN analysis after comparison to the NCBI non-redundant protein databases using BLASTX.

| **Type of**  **nucleic acids** | **Bacteria** | **Eukaryota** | **Viruses** | **Total number of reads** | **Not assigned** | **No hits** | **Number of reads assigned to taxa** | **Percentage of reads assigned to taxa** | **Percentage of reads assigned to prokaryota** |
| --- | --- | --- | --- | --- | --- | --- | --- | --- | --- |
| genomic DNA |  |  |  |  |  |  |  |  |  |
| Ir 1-4 | 122 | 10619 | 30 | 127974 | 9 | 116971 | 10994 | 8.6 | 1.1 |
| cDNA |  |  |  |  |  |  |  |  |  |
| Ir 1-4 | 179 | 2804 | 3 | 60186 | 1 | 57186 | 3015 | 5.0 | 0.3 |
