# Supplementary material for: Metagenomic Profile of the Bacterial Communities Associated with Ixodes ricinus Ticks
Source: PLoS One. 2011 Oct 13;6(10):e25604. doi: 10.1371/journal.pone.0025604 (PMC3192763; doi:10.1371/journal.pone.0025604)
Supplement: Table S3 — Properties of 454 Roche GS-FLX pyrosequencing run of the V6-16S rRNA amplicon libraries obtained from two tick pools. (DOCX) [file pone.0025604.s005.docx]

**Table S3.** Properties of 454 Roche GS-FLX pyrosequencing run of the V6-16S rRNA amplicon libraries obtained from two tick pools.

| **Amplicon 454 library** | **Type of DNA** | **Run on**  **454 GS-FLX** | **Total No.**  **of reads** | **Total base pair** | **Read average length (bp)** |
| --- | --- | --- | --- | --- | --- |
| Pool of *I. ricinus* nymphs | V6 region-16S rRNA  Primer A-B | Multiplex  on half plate | 281,327 | 54,858,765 | 194.0 |
| Pool of *I. ricinus* adults | V6 region-16S rRNA  Primer A-B | Multiplex  on half plate | 265,778 | 51,295,154 | 193.9 |
