# Supplementary material for: Metagenomic Profile of the Bacterial Communities Associated with Ixodes ricinus Ticks
Source: PLoS One. 2011 Oct 13;6(10):e25604. doi: 10.1371/journal.pone.0025604 (PMC3192763; doi:10.1371/journal.pone.0025604)
Supplement: Table S4 — Summary of the four datasets of V6-16S rRNA amplicon sequences obtained from the two tick pools after associating their geographic origin with the 5′-end barcodes. (DOCX) [file pone.0025604.s006.docx]

**Table S4**. Summary of the four datasets of V6-16S rRNA amplicon sequences obtained from the two tick pools after associating their geographic origin with the 5'-end barcodes.

| **Tick stage and No.**  **of *I. ricinus*** | **Geographic Region** | **V6 -16S rRNA reads** | **Read average length (bp)^c^** |
| --- | --- | --- | --- |
| Nymphs (N=100) | TN Region^a^ | 109,930 | 170.5 |
| Nymphs (N=100) | BL Region^b^ | 136,960 | 168.7 |
| Adults (N=20) | TN Region | 124,345 | 169.5 |
| Adults (N=20) | BL Region | 114,645 | 168.3 |

^a^ TN Region: Trento Province

^b^ BL Region: Belluno Province

^c^ Average length of the V6 amplicon reads after trimming the barcode and primer sequences
